# Mass Spectrometry-Based Solid Phase Peptide Reaction Assay for Detecting Allergenicity Using an Immobilized Peptide-Conjugating Photo-Cleavable Linker

**DOI:** 10.3390/ijms21218332

**Published:** 2020-11-06

**Authors:** Hiroshi Miyazaki, Yasutaka Samejima, Kazuya Iwata, Yuuki Minamino, Shinya Hikida, Hideto Ariumi, Hidefumi Ikeda, Yoshio Hamada, Kunihiko Yamashita, Kenji Usui

**Affiliations:** 1 Medical Device Division, Innovation and Business Development Headquarters, Daicel Corporation, Minato-ku, Tokyo 108-8230, Japan; hs_miyazaki@jp.daicel.com; 2 Faculty of Frontiers of Innovative Research in Science and Technology (FIRST), Konan University, Chuo-ku, Kobe 650-0047, Japan; s1691021@s.konan-u.ac.jp (Y.S.); s1791005@s.konan-u.ac.jp (K.I.); nan1bbmst@yahoo.co.jp (Y.M.); hshinya513@gmail.com (S.H.); pynden@gmail.com (Y.H.); 3 Faculty of Pharmaceutical Sciences, Sanyo-Onoda City University, Sanyo-Onoda, Yamaguchi 756-0884, Japan; ariumih@rs.socu.ac.jp; 4 Product Assurance Division, Mandom Corporation, Chuo-ku, Osaka 540-8530, Japan; hidefumi.ikeda@mandom.com

**Keywords:** alternative to animal testing, immobilization, peptides, skin sensitizers, mass spectrometry, photo-labile linker

## Abstract

The biological process of skin sensitization depends on the ability of a sensitizer to modify endogenous proteins. A direct peptide reactivity assay (DPRA), based on the biological process of skin sensitization, was developed as an alternative to controversial animal experiments. Although DPRA has been endorsed by industries and is internationally accepted as promising, it has several drawbacks, such as incompatibility with hydrophobic chemicals, inability to perform detailed reaction analysis, and ability to evaluate only single components. Here, we demonstrated that sensitizers and peptide adducts can be easily identified using a mass spectrometry-based solid-phase peptide reaction assay (M-SPRA). We synthesized peptides with a photo-cleavable linker immobilized on resins. We showed the potential of M-SPRA in predicting skin sensitization by measuring the peptide adducts that were selectively eluted from the resin after cleaving the linker post-reaction. M-SPRA provides more detailed information regarding chemical reactivity and accurate assessment of test samples, including mixtures. M-SPRA may be helpful for understanding the binding mechanism of sensitizers (toxicology), which may assist in further refining reactivity assays and aiding in the interpretation of reactivity data.

## 1. Introduction

The potential to trigger skin sensitization must be accurately assessed for various chemical compounds present in industrial, cosmetic, and pharmaceutical products to minimize the risk of developing skin diseases. Animal experiments, including the local lymph node assay (LLNA) [1], have been widely used to assess the sensitization potential of chemical compounds. However, animal experiments for assessing the risk potential, especially cosmetics, were prohibited in the European Union (EU) in 2011 [2]. Therefore, in vitro alternatives to animal testing for sensitization are indispensable. The biological processes underlying skin sensitization are well understood, and the covalent binding of haptens to skin proteins is a key event [3]. Based on this, a direct peptide reactivity assay (DPRA) [4,5] was developed as a promising in vitro skin sensitization test, as described in the Organization for Economic Co-operation and Development (OECD) Test Guideline 442C [6]. DPRA uses high-performance liquid chromatography (HPLC) and is based on the reactivity of a sensitizer with either cysteine (Cys) or lysine (Lys). DPRA aims to investigate the sensitizing potential of compounds by measuring the disappearance of peptides. DPRA is internationally accepted and is endorsed by industries as a promising and effective in vitro skin sensitization test. Although DPRA may be sufficient for screening purposes, the problems associated with it reduces its utility, as mentioned below [7,8,9]: (i) candidate drugs or cosmetic chemicals often have poor water solubility and thus do not dissolve in polar solvents used in the assays; (ii) the retention times of an unreacted test compound and an unreacted peptide may overlap (co-elution); (iii) the free thiol of a Cys-containing peptide can easily dimerize upon oxidation, potentially leading to false positives; (iv) use of HPLC complicates handling and is time-consuming; (v) as DPRA is an oversimplified chemical assay, specific aspects, such as reaction mechanisms, kinetics, and details of reactivity, cannot be analyzed; (vi) DPRA was developed for evaluating the skin sensitizing potential of single chemicals, because of which, information regarding the assessment of the sensitization potential of mixtures, such as essences, is negligible. In addition, the identification of sensitizers in mixtures is an essential component of product development or toxicology.

**Figure 1:**
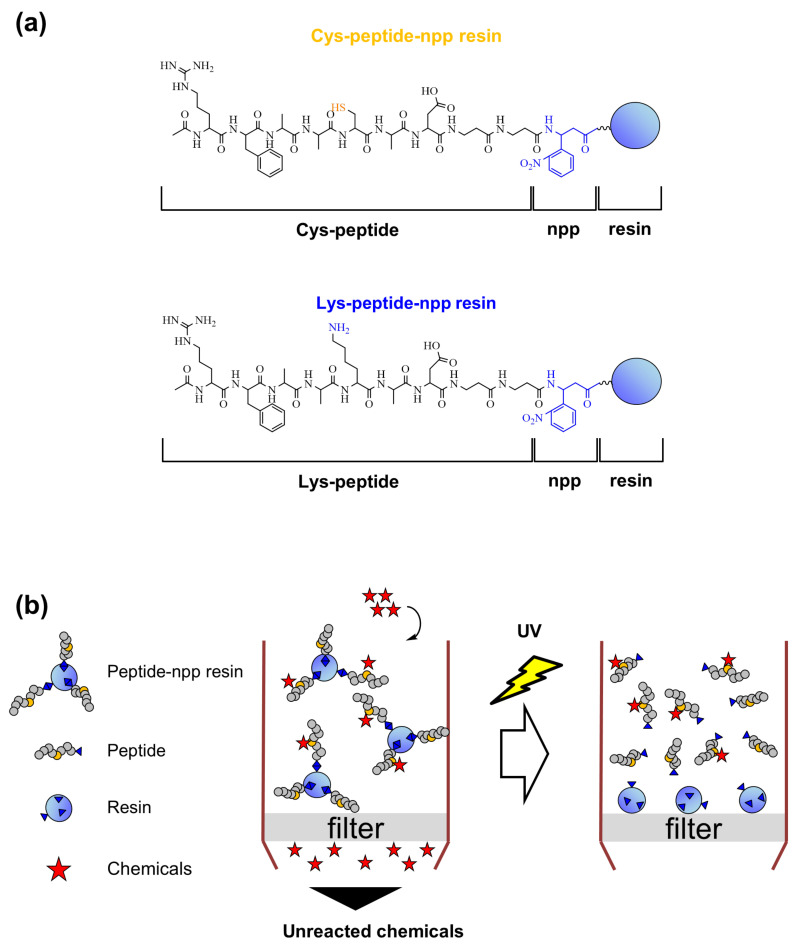
Outline of this study. (**a**) Structures of cysteine (Cys) or lysine (Lys)-peptide-npp resin. (**b**) Schematics showing the filtration of the unreacted compounds using a column and assessment of skin sensitization using a photo-cleavable linker and solid phase-immobilized peptides.

In our previous studies, we have developed an in vitro chromophore-solid phase peptide reaction assay (C-SPRA) using solid phase-immobilized peptides and chromophores and reported that the use of immobilized peptides can overcome the limitations of DPRA [10]. Furthermore, in this study, we demonstrated that the combination of a photo-cleavable linker and solid phase-immobilized peptides provides more detailed information regarding the chemical reactivity and binding mechanism of sensitizers and enables accurate assessment of test samples, including mixtures, than the previous method (Figure 1). Ortho-nitrobenzylamine derivatives are rapidly cleaved after irradiation with UV light at 365 nm [11,12,13,14,15,16,17] and are widely used as cleavable linkers under mild conditions. Sensitizers can be bound to the peptide via a photo-cleavable linker on the resin, and only the sensitizers that react with peptides are selectively eluted from the resin by cleaving the linker. We studied whether the sensitizer and peptide adduct can be easily identified using a mass spectrometry (MS)-based solid-phase peptide reaction assay (M-SPRA). Furthermore, we investigated whether M-SPRA can be used to identify the sensitizer in a mixture that binds to the peptide.

## 2. Results and Discussion

### 2.1. Design and Synthesis of Cys- and Lys-Peptides via a Photo-Cleavable Linker on the Resin

We designed and synthesized peptides with a photo-cleavable linker immobilized on resins (Cys- and Lys-peptide-npp resin) (Figure 1a). The TentaGel S NH_2_ resin was selected, as it is an NH_2_–PEG(polyethylene glycol)–polystyrene resin and swells in both hydrophilic and hydrophobic solvents. DPRA peptides (Cys- and Lys-peptide) were synthesized via a photo-cleavable npp (3-amino-3-(2′-nitrophenyl) propionic acid) linker and, we immobilized the carboxyl group of the peptide and the *N*-terminus of the amphiphilic TentaGel S NH_2_ resin after optimization of the Fmoc peptide synthesis protocol [14,18]. Studies have shown that the photo-cleavable npp linker is rapidly cleaved by irradiation at 365 nm under mild conditions without additives and is widely used in solid-phase peptide synthesis [14,15,16] (Figure 1b and Figure S1a). For checking the photo-cleaving and the purity, Cys- and Lys-peptide-npp resins in a tube were irradiated with UV light, and then, the solution was analyzed by HPLC and matrix-assisted laser desorption ionization-time of flight (MALDI-TOF) MS. These results showed that the photo-cleaved peptides were sufficient in purity and amounts for the following experiments (Figure S1b–e).

Using these peptide resins, the assay can be started easily by adding the solvent, including test samples. Especially, poorly water-soluble samples can be solved in organic (hydrophobic) solvents, and the reaction can be started. Then, the reaction can be stopped easily by washing the peptide-npp resin, and detailed reactivity can be evaluated using MALDI-TOF MS.

### 2.2. Optimization of MALDI-TOF MS Analysis Using FITC-Modified Peptides

**Figure 2:**
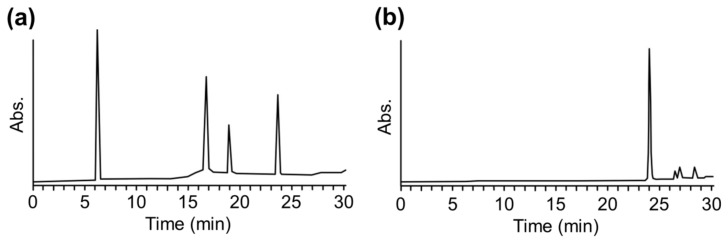
(**a**) High-performance liquid chromatography (HPLC) chromatographs of peptide adducts after reaction of fluorescein-5-isothiocyanate (FITC) with the Lys-peptide-npp resin. (**b**) HPLC chromatographs of only FITC. HPLC was performed using an Inertsil C4 column (10.0 × 250 mm) for analysis with a linear gradient from 0% to 100% of solvent B (95% acetonitrile, 5% MilliQ water, and 0.08% TFA) over 30 min at a flow rate of 3.0 mL/min. The eluate was monitored at 220 nm.

We optimized the assay protocols using Lys-peptide-npp resin and fluorescein-5-isothiocyanate (FITC) as a representative test chemical by checking HPLC. For the reaction of Lys-peptide-npp resin and 100 mM FITC and subsequent photo-cleavage, the Lys-peptide adduct was assessed using HPLC by monitoring the absorbance of the peptide moiety at 220 nm. The HPLC chromatogram showed a peak at 19 min, corresponding to the Lys-peptide adduct (Figure 2a). The HPLC chromatogram of only 50 μM FITC is shown in Figure 2b, which showed a peak at 24 min, corresponding to FITC. The concentration of FITC after the peptide-npp-resin wash was lower than 50 µM, which was 2000 times or lesser than the reaction concentration. These results showed that the unreacted chemicals can be easily removed from the peptide-npp-resin via washing and that only the peptide adduct is released from the resin. Using the above approach, we obtained data regarding the reactivity of the Cys-, Lys-peptide-npp resin, and sensitizers.

Then, MALDI-TOF MS analysis of FITC-modified peptide was conducted. The reaction of Lys-peptide-npp resin and 100 mM FITC and subsequent photo-cleavage, the Lys-peptide adduct, was assessed using MALDI-TOF MS. The MS showed an m/z peak at 1279, corresponding to the Lys-peptide adduct (Figure S2). Additionally, we diluted the cleaved FITC-modified peptide solution at various times and assessed them using MALDI-TOF MS to check MS detection limit (Figure S3). The results showed that even 2000 times dilution sample (50 µM) provided the desired adduct peak. Then, to confirm whether a small amount of sensitizer in the mixture can be evaluated, a reaction was performed at low concentration and high volume. Lys-peptide adducts were observed when the Lys-peptide-npp resin peptide was incubated with 50 mL of 25 µM FITC under the MS detection limit concentration, indicating that even low concentrations of sensitizers can be supplemented with M-SPRA (Figure S4). In the conventional analysis, DPRA, for assessment, 100 mM of the test sample should be applied. Consequently, we selected 100 mM of sample concentration as a standard in the following experiments.

### 2.3. MALDI-TOF MS Analysis of Sensitizer-Modified Peptides

The Cys- and Lys-peptides that reacted with the sensitizers were analyzed using MALDI-TOF MS to confirm the formation of peptide adducts (Table 1). Whether the chemicals formed adducts was determined from the peak of the peptide signal, which indicated that the chemical substance was clearly reactive. The signals of Cys- and Lys-peptides were observed at *m*/*z* = 865 and 890 ([M+H]^+^ of the peptide). In the case of Cys-peptide, the appearance of new signals could not be explained by peptide oxidation and were considered adducts. The peak at *m*/*z* = 1727 ([M+H]^+^ of the peptide dimer) was consistent with the formation of an oxidized Cys-peptide dimer. All sensitizers tested were observed for either Cys- or Lys-peptide adduct using M-SPRA. No peptide adducts were observed with the tested non-sensitizers (dibutyl phthalate (DP) and isopropanol (IPA)), which were similar to the results obtained using DPRA. The case of undec-10-enal (UE), α-hexyl cinnamic aldehyde (HCA), α-amyl cinnamic aldehyde (ACA), and benzyl cinnamate (BC) are also worth mentioning, as significant peptide depletion ratio in DPRA was not observed for these chemicals, although a clear Cys- or Lys-peptide adduct was identified. This indicated that M-SPRA may be more sensitive than DPRA. However, the reason behind the increased depletion ratio in DPRA in the absence of dimer or adduct peak requires further investigation. Furthermore, these results suggested that immobilized peptides in M-SPRA, as well as C-SPRA allow the hydrophobic solvent to be used in the skin sensitizer tests for the assessments of poorly water-soluble chemicals that DPRA is unable to assess. Additionally, this implies that our method offers easy handling and general versatility, as well as higher reproducibility and accuracy than DPRA.

Benzylidene acetone (BA) has been reported as a sensitizer by human data, such as HMT and HRIPT. ACA and HCA are classified as weak sensitizers according to LLNA and are considered inconclusive results in the human data. Furthermore, IP has been reported as a non-sensitizer by the human data. These results were correlated with M-SPRA results. These results imply that M-SPRA can accurately predict sensitizers judged to be false negatives or inconclusive results by DPRA and the human data.

### 2.4. Confirmation of Reactivity with Adduct Analysis

Adduct analyses using mass analysis provided additional information regarding reactivity that is not apparent from the peptide depletion ratio of DPRA. The majority of the adducts observed resulted from reactions involving a single and often easily predicted mechanism. The novel peaks for each test chemical are summarized in Table 2.

Along with the m/z of the observed base ions, a hypothetical explanation for each of the adduct peaks is indicated in Table 2. For many chemicals, the molecular weight of the novel peak indicated the formation of the Michael adduct or Schiff base, which can be easily explained by the addition–elimination reaction. Michael adducts containing the Cys-peptide and Schiff base containing the Lys-peptide were observed with various sensitizing chemicals. In addition, several examples of complex reactivity, such as the generation of multiple adducts and multiple reaction mechanisms, were observed. *p*-Benzoquinone (BQ) formed peptide adducts with two molecules of the test chemical, which were added to different nucleophilic residues within the same peptide. Complex cross-linked structures were observed in the reaction of Lys and *N*-term peptides with BQ [20]. It is currently not clear how complex reactivity may generally affect the sensitization potency of chemicals. We presented a more detailed structural analysis of the adduct but only provided a hypothetical interpretation of the adduct ions. However, a novel adduct with a test peptide is a sufficient indicator for identifying a sensitizer, even if the exact structure is unknown.

The MS assay is a useful tool that can replace HPLC for the detection of peptide depletion, with the possibility of adduct formation being the most important improvement. While analysis of the average depletion values using DPRA provides simple measurements that are generally acceptable, M-SPRA provides a more detailed analysis than DPRA and produces large amounts of data. In particular, for chemicals belonging to the Michael acceptor or Schiff base reactivity domains, adducts of the predicted molecular weight were detected; thus, MS analysis corroborated the tentative structure-based attribution to reactivity domains [21,22,23]. Structural elucidation will be an important step for mechanistic understanding and will be especially helpful for the optimization of products. In addition, it is likely that a thorough understanding of all aspects of chemical reactivity may assist in rationalizing the toxicological effects. M-SPRA can be combined with the previously reported C-SPRA [10] to allow further detailed reactivity, including kinetics and reaction mechanisms because it is possible to stop the reaction easily by washing in SPRA.

### 2.5. Reaction of Mixture with the Peptide-npp Resin

**Figure 3:**
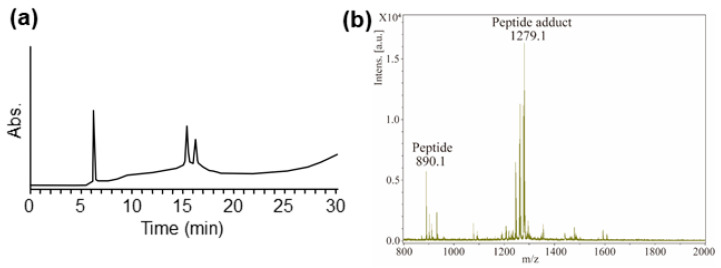
(**a**) HPLC chromatographs of peptide adducts after reaction of a mixture of FITC- isopropanol (IPA) with Lys-peptide-npp resin. (**b**) Mass spectrum at the retention time of 15–16 min in HPLC from Figure 3a.

Adducts were observed using mixtures to assess the applicability of M-SPRA to the mixtures. Two simple combinations (FITC-IPA, BA-IPA, and ACA-IPA) were selected as the mixture. The characterized adducts were in accordance with those observed when tested separately. Figure 3a shows an example of the results of HPLC for the peptide, the direct adduct observed after incubation with FITC-IPA. The characterized adducts are shown in Figure 3b. When the FITC-IPA mixture was mixed with the peptide, only the adduct with FITC was observed, whereas reaction with IPA was not observed. The results showed that the sensitizer in mixtures can react with the peptides and that M-SPRA may be applicable to the mixture. Thus, M-SPRA is also applicable to cases where it is necessary to assess the reactivity of chemicals in mixtures. The interaction between the components in the mixture may affect the overall chemical reactivity. Some sensitizers are not directly reactive and require some form of activation either via spontaneous oxidation on air exposure or metabolic activation in the epidermis [24,25].

## 3. Materials and Methods

### 3.1. Test Chemicals and Materials

The test chemicals were BQ, FITC, BA, UE, MPH, HCA, ACA, BC, DP, and IPA. BQ, BA, UE, HCA, BC, and IPA were purchased from FUJIFILM Wako Pure Chemical Corporation (Wako, Osaka, Japan). FITC was purchased from AdipoGen Life Sciences, Inc. (San Diego, CA, USA). MPH and ACA were purchased from Tokyo Chemical Industry Co., Ltd. (Tokyo, Japan). DP was purchased from Sigma–Aldrich Co. LLC (St. Louis, MO, USA). The test chemicals were dissolved in Peptide synthesis grade *N*,*N*-dimethyl formamide (DMF) (Wako) and *N*-methylmorpholine (Wako). All chemicals were of special grade and were used without further purification.

The amino acid monomers, *N*-α-Fmoc-alanine (Fmoc-Ala-OH), *N*-β-Fmoc-alanine (Fmoc-βAla-OH), *N*-α-Fmoc-*N*-ε-Boc-lysine (Fmoc-Lys(Boc)-OH), *N*-α-Fmoc-S-trityl-cysteine (Fmoc-Cys(Trt)-OH), and *N*-α-Fmoc-phenylalanine (Fmoc-Phe-OH), *N*-α-Fmoc-NG-(2,2,4,6,7-pentamethyldihydrobenzofuran-5-sulfonyl)-arginine (Fmoc-Arg(Pbf)-OH), *N*-α-Fmoc-aspartic acid β-t-butyl ester (Fmoc-Asp(OtBu)-OH), and Fmoc-3-amino-3-(2′-nitrophenyl) propionic acid (Fmoc-npp-OH) were used. All Fmoc amino acids were purchased from HiPep Laboratories (Kyoto, Japan). The condensation reagents, 1-[bis(dimethylamino)methylene]-1H-1,2,3-triazolo[4,5-b]pyridinium 3-oxide hexafluorophosphate (HATU), [2-(1H-benzotriazole-1-yl)-1,1,3,3-tetramethyluronium hexafluorophosphate (HBTU), and 1-hydroxy benzotriazole monohydrate (HOBt) were purchased from Watanabe Chemical Industries, Ltd. (Hiroshima, Japan). Trifluoroacetic acid (TFA) was purchased from Watanabe Chemical Industries, and triisopropylsilane was purchased from Wako. All aqueous solutions were prepared using distilled/deionized water.

### 3.2. Synthesis of Cys-Peptide-npp and Lys-Peptide-npp Resins

Cys-peptide-npp and Lys-peptide-npp resins were synthesized on TentaGel S NH_2_ resin (HiPep Laboratories) via manual synthesis using Fmoc chemistry [26]. Coupling of Fmoc-npp-OH and Fmoc-Asp(OtBu)-OH was performed using HATU (4 eq.), and coupling of Fmoc-βAla-OH, Fmoc-Ala-OH, Fmoc-Lys(Boc)-OH, Fmoc-Cys(Trt)-OH, Fmoc-Phe-OH, and Fmoc-Arg(Pbf)-OH were performed using HBTU and HOBt (10 eq.). Initially, Fmoc-npp-OH was coupled to the resins, and then two peptides containing either Cys or Lys (Ac-RFAACADβA-npp-resin and Ac-RFAACADβA-npp-resin) were synthesized. The side-chain protecting groups on the synthesized peptide resins were removed by incubating the resins for 2 h in a deprotection solution (TFA/H_2_O/triisopropylsilane (50:1:1, *v*/*v*)). The resins were washed five times, each with a washing solution (TFA/H_2_O (100:1)), *N*-methylpyrrolidone, and chloroform, and then dried completely in a desiccator.

### 3.3. Procedure Using Cys-Peptide-npp Resins

Cys-peptide-npp resins (1 mg dry resin) were placed in two test tubes and swelled for 24 h with 0.3 mL of 0.1 mM dithiothreitol (DTT) (Wako) solution (in DMF). The swollen resins were washed five times with DMF. One test tube was incubated with 1 mL of 100 mM test compound solution (in DMF) at room temperature (20–25 °C) for 24 h (test). The other test tube was incubated in DMF at room temperature for 24 h (control). After incubation, the resins were washed once with DMF and then five times with H_2_O/acetonitrile/TFA (95:5:0.01, *v*/*v*). Cys-peptide-npp resins were irradiated with UV light for 20 min to cleave the peptide from the resins in 300 µL of the cleavage solution (95% MilliQ water, 5% acetonitrile, and 0.01% TFA). Then the absorbed substances on the resin were washed out by 300 µL of the wash solution (50% acetonitrile, 50% MilliQ water). Both the cleavage solution and the wash solution were analyzed using MALDI-TOF MS or HPLC.

### 3.4. Procedure Using Lys-Peptide-npp Resins

Lys-peptide-npp resins (1 mg as dry resin) were placed in two test tubes and swelled for 24 h with 0.3 mL DMF. The swollen resins were washed five times with DMF. One test tube was incubated with 1 mL 100 mM test compound solution (in DMF containing 1% *N*-methylmorphiline) at room temperature for 24 h (test). The other test tube was incubated in DMF at room temperature for 24 h (control). After incubation, the resins were washed once with DMF and then five times with H_2_O/acetonitrile/TFA (95:5:0.01, *v*/*v*). To cleave the peptide from the resins, Lys-peptide-npp resin was irradiated with UV light for 20 min in 300 µL of the cleavage solution (95% MilliQ water, 5% acetonitrile, and 0.01% TFA). Then the absorbed substances on the resin were washed out by 300 µL of the wash solution (50% acetonitrile, 50% MilliQ water). Both the cleavage solution and the wash solution were analyzed using MALDI-TOF MS or HPLC.

### 3.5. Analysis Using HPLC

The HPLC was performed on the Alliance HPLC system (e2695 separation module and 2489 detector) (Nihon Waters K.K., Tokyo, Japan) using an Inertsil C4 column (10.0 × 250 mm) for analysis with a linear gradient from 0% to 100% of solvent B (95% acetonitrile, 5% MilliQ water, and 0.08% TFA) and solvent A (95% MilliQ water, 5% acetonitrile, and 0.1% TFA) over 30 min at a flow rate of 3.0 mL/min, or using an Inertsil ODS-3 column (4.5 × 150 mm; GL Science), and a linear acetonitrile/0.1% TFA gradient at a flow rate of 1.0 mL/min. The eluate was monitored at 220 nm and 254 nm.

### 3.6. Analysis Using MALDI-TOF MS

The peptide solution cleaved from the resins (the cleavage solution and the wash solution in 3.3 and 3.4) was analyzed using MALDI-TOF MS on an Autoflex III mass spectrometer (Bruker Daltonics, Billerica, MA, USA) with 3,5-dimethoxy-4-hydroxycinnamic acid as the matrix. The TOF data were externally calibrated using angiotensin II and insulin as standards.

## 4. Conclusions

We demonstrated that sensitizers and peptide adducts can be easily identified using M-SPRA, and that this approach can be used for evaluating chemicals with diverse skin sensitization potentials and mixtures, including sensitizers. Our results suggested that compared to DPRA, M-SPRA may be useful for screening compounds by accurately predicting the possibility of skin sensitization. Immobilized peptides allow the hydrophobic solvent to be used in the skin sensitizer tests for the assessments of poorly water-soluble chemicals that DPRA is unable to assess. In addition, using immobilized peptides, the unreacted chemical can be removed at any point by washing, and the mixture can be easily evaluated, and it is possible to stop the reaction easily by washing and to evaluate the reactivity kinetically. This system using immobilized peptide holds promise as a next-generation in vitro skin sensitization test that can address the disadvantages of DPRA. Moreover, this system can be combined with C-SPRA [10], allowing simultaneous quantitative and qualitative analyses to improve the assay throughput. This platform may provide further detailed information regarding the reaction mechanism. This study expands on the previously developed C-SPRA for assessing skin sensitizers without using animals for testing in food, biochemical, medicinal, and cosmetic engineering. By measuring the reactivities of various chemicals using M-SPRA, we may better understand the process of skin sensitization (toxicology) at the molecular level, which may also contribute to developments in the field of biochemical engineering.

## Figures and Tables

**Table 1:** Summary of signals for the peptide adducts.

Test Chemicals	LLNA ^a^	DPRA ^b^	Adduct Formation in this Study ^c^	
	Potency Category	Results ^d^	Cys-Peptide	Lys-Peptide
*p*-Benzoquinone (BQ)	Extreme	P	+	+
Fluorescein-5-isothiocyanate (FITC)	Strong	P	+	+
Benzylidene acetone (BA)	Moderate	P	+	+
5-Methyl-2-phenyl-2-hexenal (MPH)	Moderate	-	+	+
Undec-10-enal (UE)	Moderate	N	−	+
α-Hexyl cinnamic aldehyde (HCA)	Weak	N	+	+
α-Amyl cinnamic aldehyde (ACA)	Weak	N	+	+
Benzyl cinnamate (BC)	Weak	N	+	−
Dibutyl phthalate (DP)	Non-sensitizer	N	−	−
Isopropanol (IP)	Non-sensitizer	N	−	−

^a^ Local lymph node assay. ^b^ Direct peptide reactivity assay.^c^ Changes in the mass signal after reaction of peptides with chemicals were used to distinguish between ‘+’ (adduct formation) and ‘−’ (no adduct formation). ^d^ Data from Otsubo et al., 2017 [19].

**Table 2:** Summary of detailed information on peptide adducts.

Test Chemicals	MS Signal (*m*/*z*)		Adduct Interpretation
	Cys-Peptide	Lys-Peptide	
BQ	Cys1: 973 [M+H]^+^ Cys2: 1113 [M+H]^+^	Lys1: 1101 [M+H]^+^	Cys1: Michael adduct, Cys2: bimolecular addition by Michael adduct and oxidation to sulfone, Lys1: bimolecular addition by Michael adduct
FITC	Cys1: 1254 [M+H]^+^	Lys1: 1279 [M+H]^+^	Cys1: Acylation, Lys1: Acylation
BA	Cys1: 1011 [M+H]^+^	Lys1: 1164 [M+H]^+^	Cys1: Michael adduct, Lys1: bimolecular addition by Michael adduct and Schiff base formation
MPH	Cys1: 1053 [M+H]^+^	Lys1: 1074 [M+H]^+^	Cys1: Michael adduct, Lys1: unknown
UE	No signal	Lys1: 1042 [M+H]^+^	Lys1: Schiff base formation
HCA	Cys1: 1081 [M+H]^+^	Lys1: 1120 [M+H]^+^	Cys1: Michael adduct, Lys1: Michael adduct
ACA	Cys1: 1067 [M+H]^+^	Lys1: 1074 [M+H]^+^	Cys1: Michael adduct, Lys1: Schiff base formation
BC	Cys1: 1085 [M+H]^+^ Cys2: 1131 [M+H]^+^	No signal	Cys1: unknown, Cys2: unknown,
DP	No signal	No signal	Not adduct
IP	No signal	No signal	Not adduct

## Supplementary Materials

The Supplementary Materials are available online at https://www.mdpi.com/1422-0067/21/21/8332/s1.

## Abbreviations

ACA	a-Amyl cinnamic aldehyde
BA	Benzylidene acetone
BC	Benzyl cinnamate
BQ	*p*-Benzoquinone
DMF	Dimethyl formamide
DP	Dibutyl phthalate
DPRA	Direct peptide reactivity assay
FITC	Fluorescein-5-isothiocyanate
HCA	a-Hexyl cinnamic aldehyde
HPLC	High-performance liquid chromatography
IPA	Isopropanol
MS	Mass spectrometry
MPH	5-Methyl-2-phenyl-2-hexenal
M-SPRA	Mass spectrometry-based solid-phase peptide reaction assay
UE	Undec-10-enal
