# Supplementary material for: Mass Spectrometry-Based Solid Phase Peptide Reaction Assay for Detecting Allergenicity Using an Immobilized Peptide-Conjugating Photo-Cleavable Linker
Source: Int J Mol Sci. 2020 Nov 6;21(21):8332. doi: 10.3390/ijms21218332 (PMC7664224; doi:10.3390/ijms21218332)
Supplement: Supplementary file 1 [file ijms-21-08332-s001.pdf]

International Journal of *Molecular Sciences*

MDPI

Supplementary Materials for

Mass Spectrometry-Based Solid Phase Peptide Reaction Assay for Detecting Allergenicity Using an Immobilized Peptide-Conjugating Photo-Cleavable Linker

Hiroshi Miyazaki [1], Yasutaka Samejima [2], Kazuya Iwata [2], Yuuki Minamino [2], Shinya Hikida [2], Hideto Ariumi [3], Hidefumi Ikeda [4], Yoshio Hamada [2], Kunihiko Yamashita [1,*] and Kenji Usui [2,*]

[1] Medical Device Division, Innovation and Business Development Headquarters, Daicel Corporation, Minato-ku, Tokyo 108-8230, Japan; hs_miyazaki@jp.daicel.com

[2] Faculty of Frontiers of Innovative Research in Science and Technology (FIRST), Konan University, Chuo-ku, Kobe 650-0047, Japan; s1691021@s.konan-u.ac.jp (Y.S.); s1791005@s.konan-u.ac.jp (K.I.); nan1bbmst@yahoo.co.jp (Y.M.); hshinya513@gmail.com (S.H.); pynden@gmail.com (Y.H.)

[3] Faculty of Pharmaceutical Sciences, Sanyo-Onoda City University, Sanyo-Onoda, Yamaguchi 756-0884, Japan; ariumih@rs.socu.ac.jp

[4] Product Assurance Division, Mandom Corporation, Chuo-ku, Osaka 540-8530, Japan; hidefumi.ikeda@mandom.com

* Correspondence: ku_yamashita@jp.daicel.com (K.Y.); kusui@konan-u.ac.jp (K.U.); Tel.: +81-6-6210-8449 (K.Y.); +81-78-303-1418(K.U.)

Table of Contents

Page 1: Contents

Pages 2-3: Figure S1 for scheme of the photo cleavage and, HPLC chart and mass spectra of peptide-npp resins.

Page 4: Figure S2 for mass spectrum after the 100 mM FITC assay using Lys-peptide-npp resin.

Pages 5-7: Figure S3 for mass spectra of diluted cleavage solution after the 100 mM FITC reaction using Lys-peptide-npp resin.

Page 8: Figure S4 for mass spectrum after the reaction with 50 mL of 25 µM FITC using Lys-peptide-npp resin.

(a)

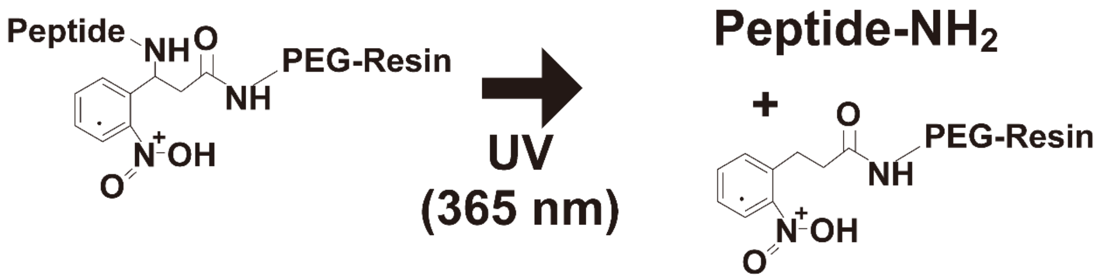
Peptide
NH
O
PEG-Resin
NH
N+ -OH
O
UV
(365 nm)
Peptide-NH2
+
O
PEG-Resin
NH
N+ -OH
O

(b)

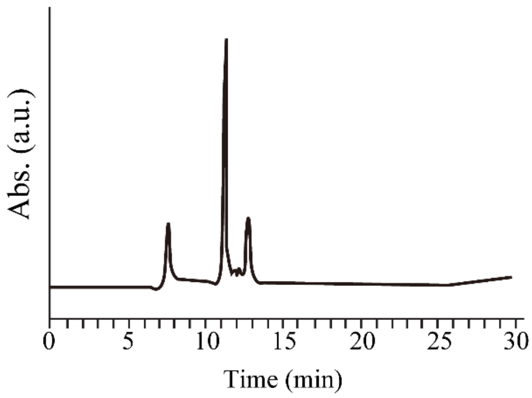
Abs. (a.u.)
0
5
10
15
20
25
30
Time (min)

(c)

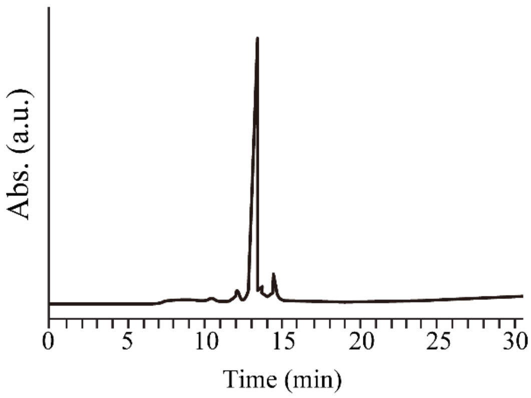
Abs. (a.u.)
0
5
10
15
20
25
30
Time (min)

(d)

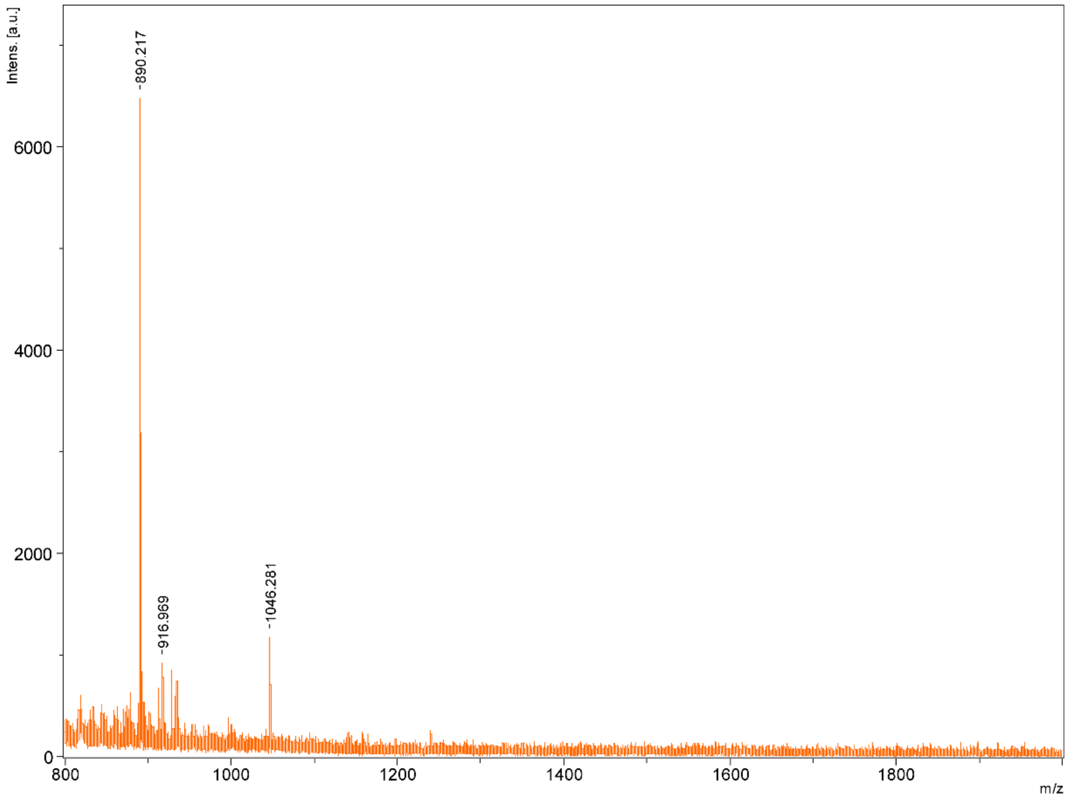
Intens. [a.u.]
890.217
916.969
1046.281
6000
4000
2000
0
800
1000
1200
1400
1600
1800
m/z

(e)

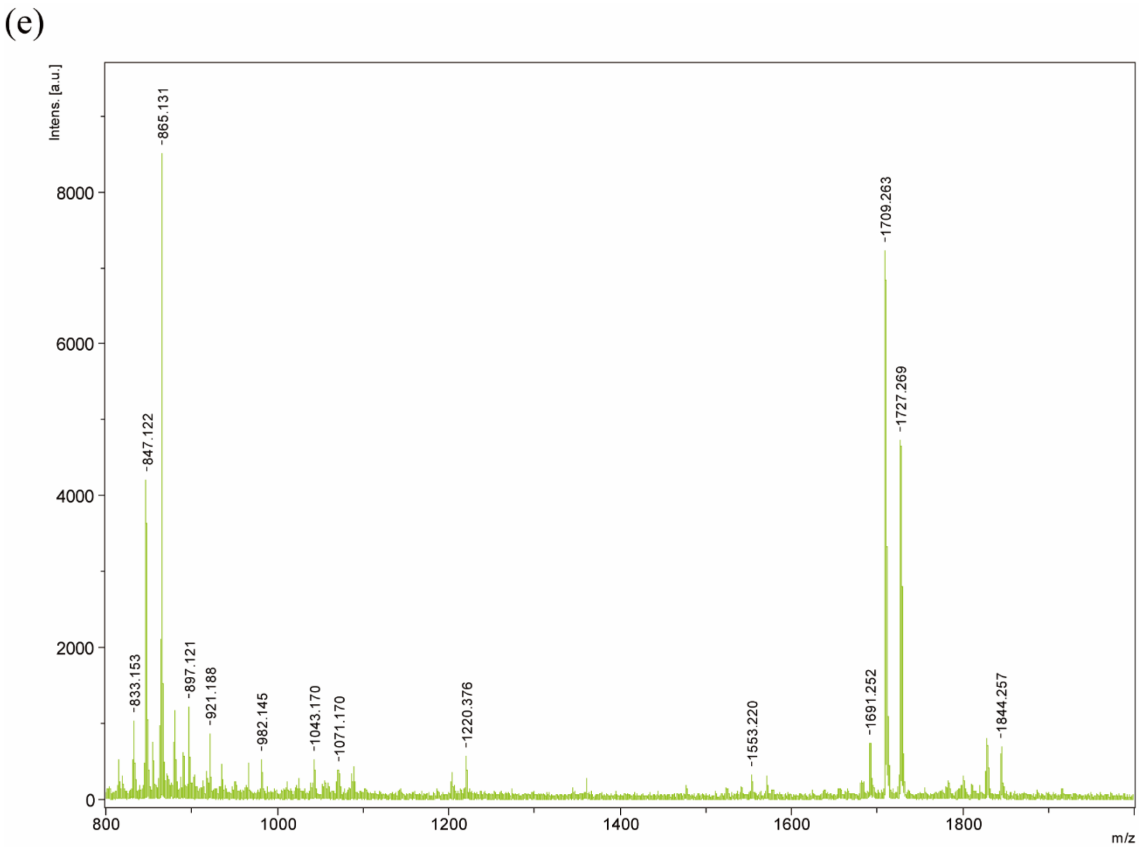

Figure S1 (a) Scheme of the photo cleavage in this study. (b) HPLC chromatographs of the cleaved peptide from the Lys-peptide-npp resin. (c) HPLC chromatographs of the cleaved peptide from the Cys-peptide-npp resin. (d) Mass spectrum after the cleavage of the peptide from Lys-peptide-npp resin. (e) Mass spectrum after the cleavage of the peptide from Cys-peptide-npp resin. The HPLC was performed using an Inertsil ODS-3 column. The eluate was monitored at 220 nm.

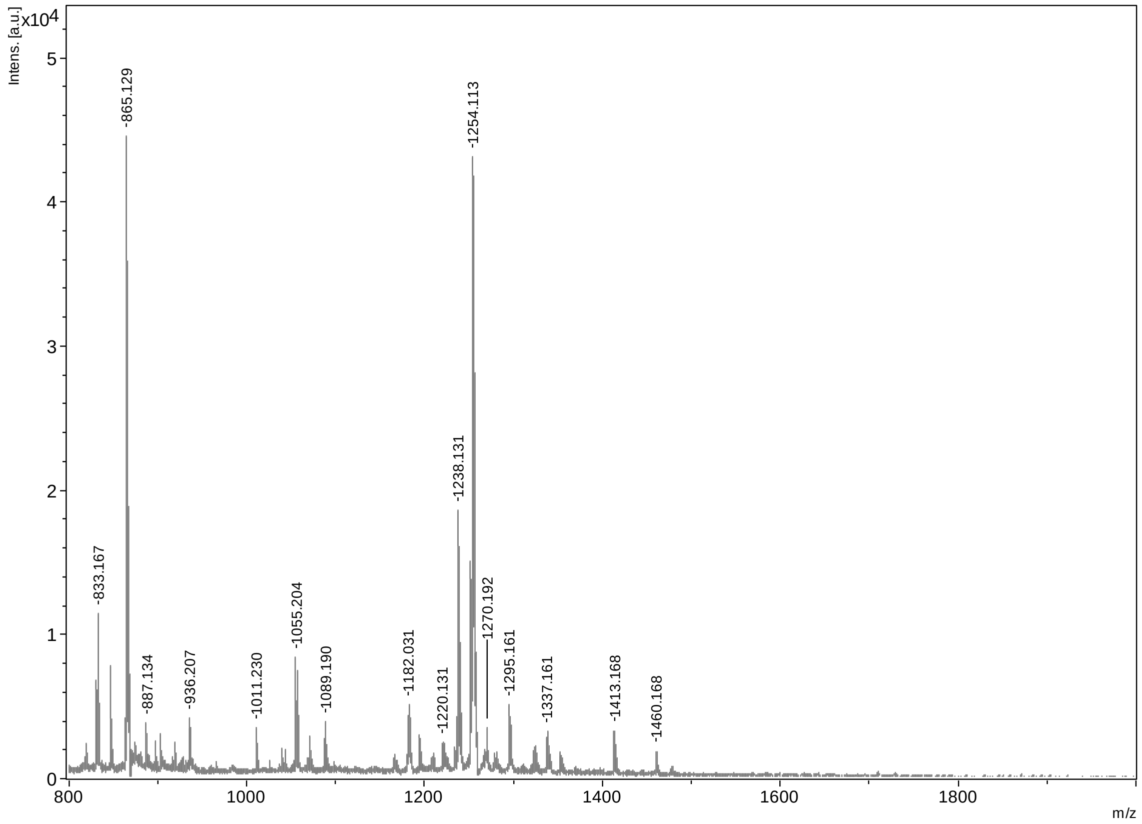

Figure S2. Mass spectrum after the 100 mM FITC assay using Lys-peptide-npp resin.

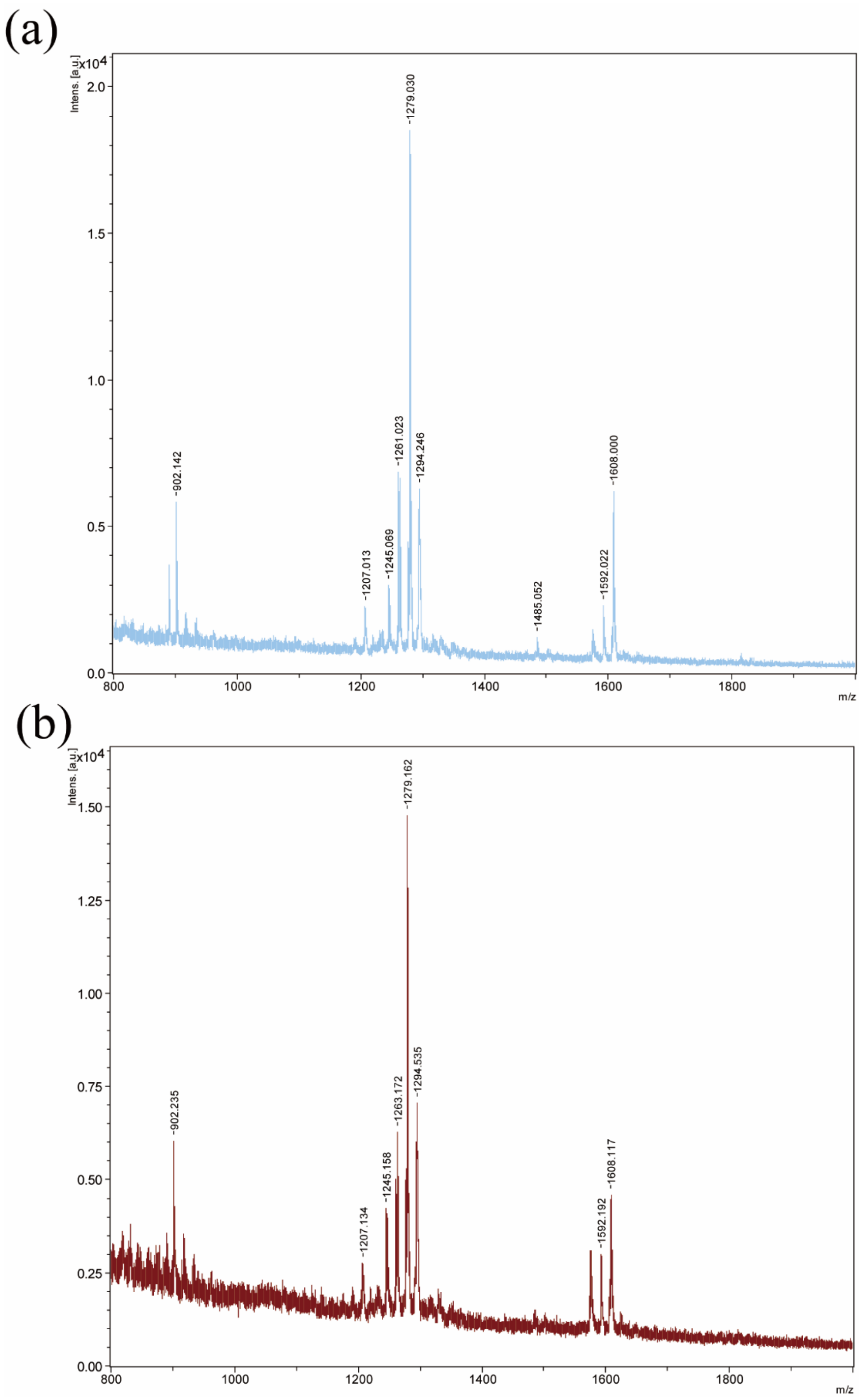
(a)
Intens. [a.u.]
x10^4
2.0
1.5
1.0
0.5
0.0
902.142
1207.013
1245.069
1261.023
1279.030
1294.246
1485.052
1592.022
1608.000
800
1000
1200
1400
1600
1800
m/z
(b)
Intens. [a.u.]
x10^4
1.50
1.25
1.00
0.75
0.50
0.25
0.00
902.235
1207.134
1245.158
1263.172
1279.162
1294.535
1592.192
1608.117
800
1000
1200
1400
1600
1800
m/z

(c)

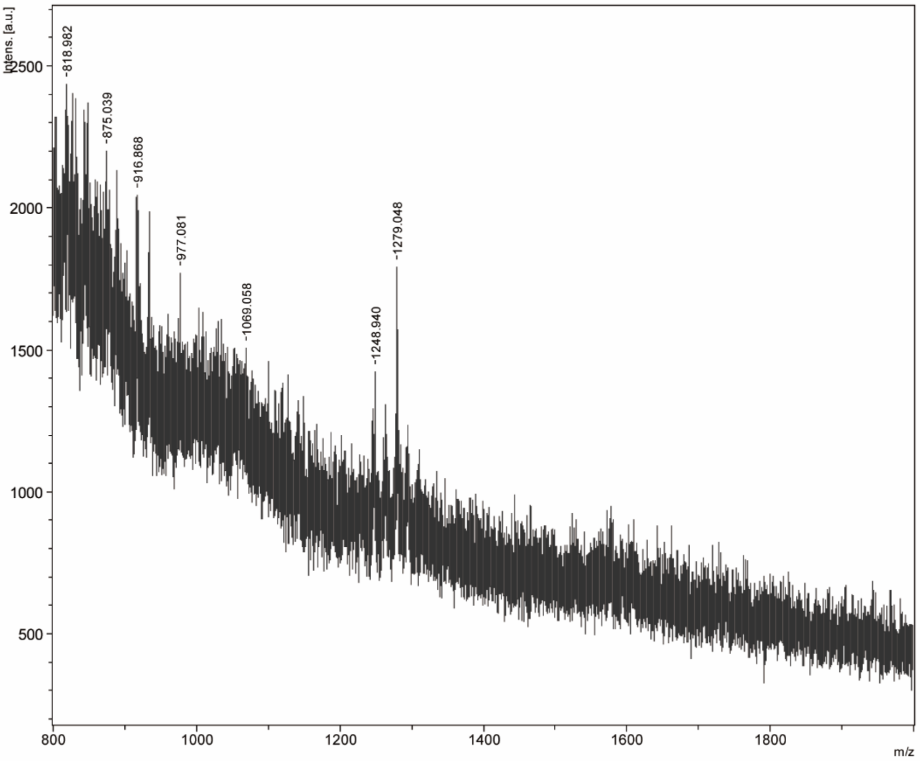
Intens. [a.u.]
818.982
875.039
916.868
977.081
1069.058
1248.940
1279.048
2500
2000
1500
1000
500
800
1000
1200
1400
1600
1800
m/z

(d)

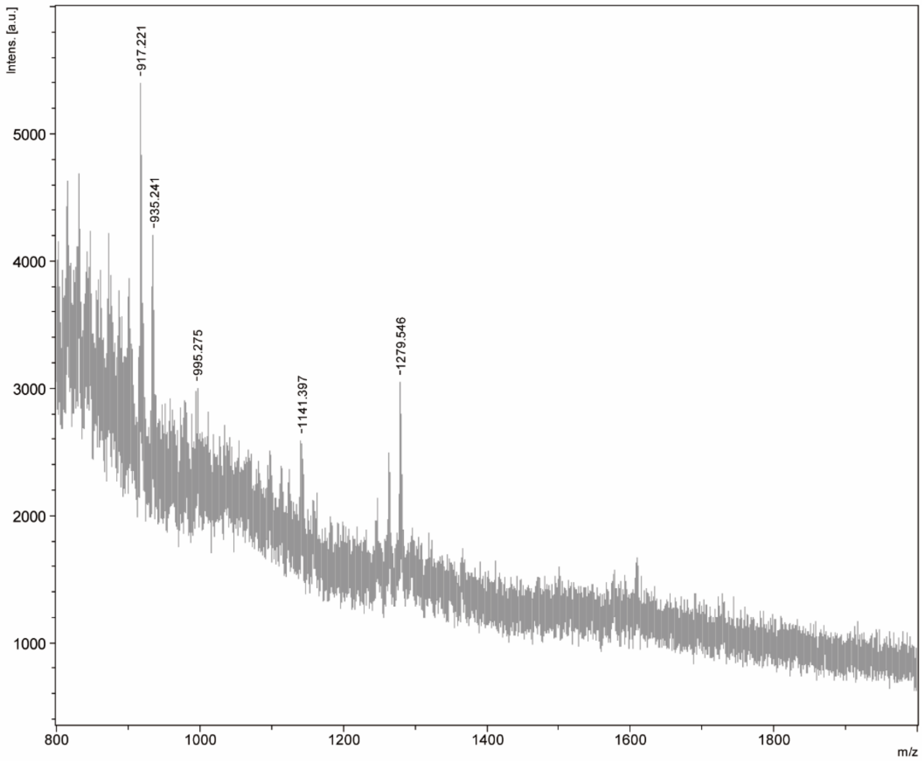
Intens. [a.u.]
917.221
935.241
995.275
1141.397
1279.546
5000
4000
3000
2000
1000
800
1000
1200
1400
1600
1800
m/z

(e)

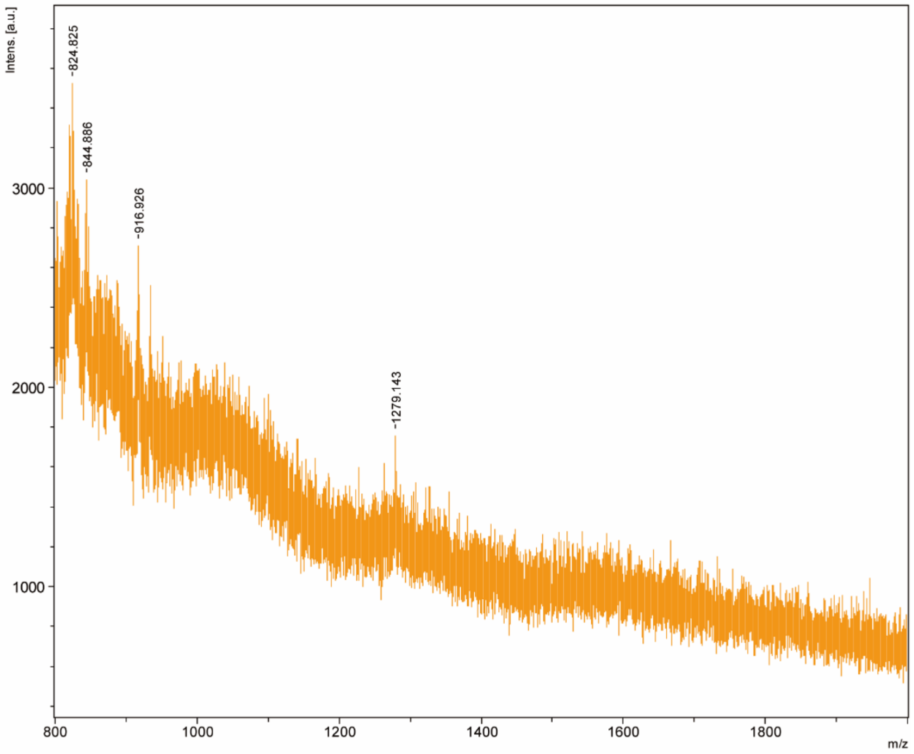

(f)

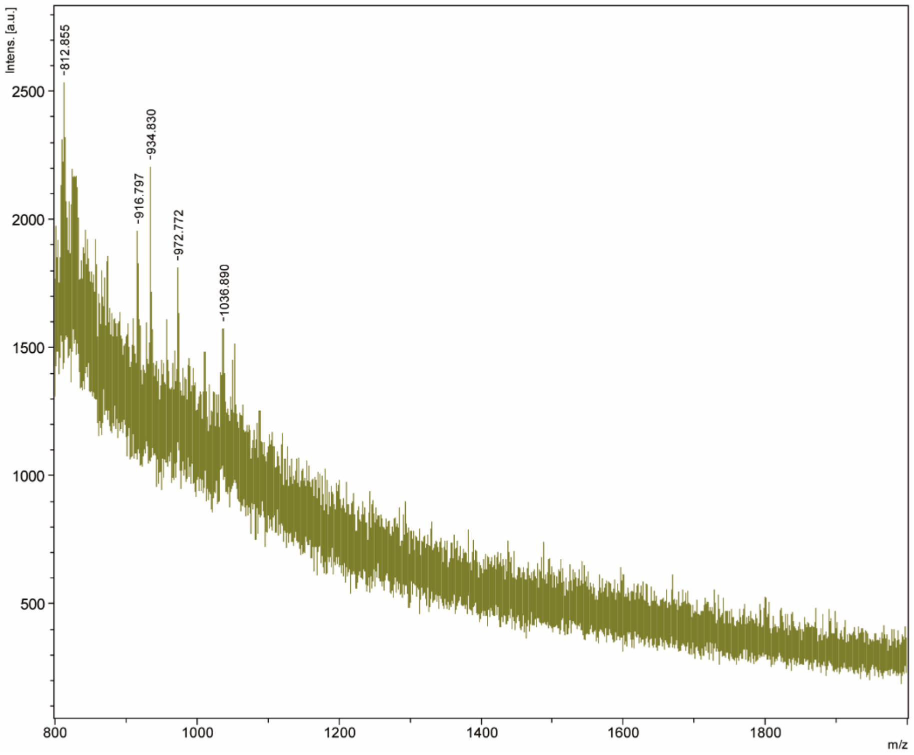

Figure S3. Mass spectra of diluted cleavage solution in various concentrations ((a) 10 mM, (b) 1 mM, (c) 100 μM, (d) 75 μM, (e) 50 μM, (f)25 μM) after the 100 mM FITC reaction using Lys-peptide-npp resin.

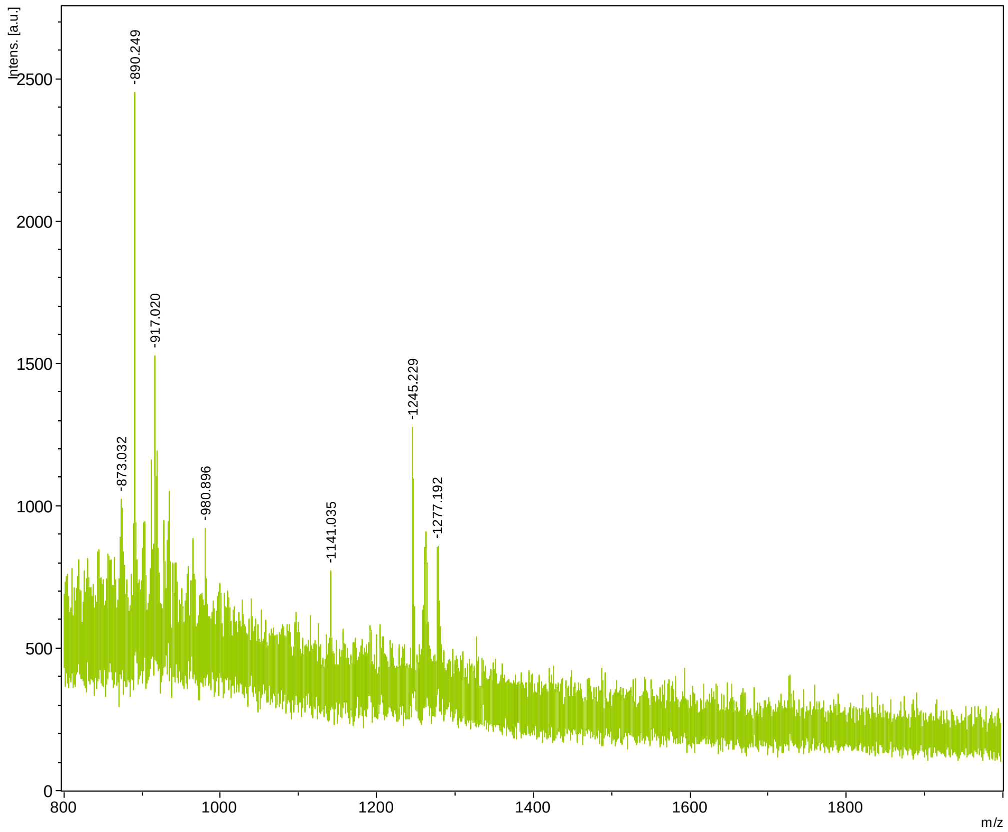

Figure S4. Mass spectrum after the reaction with 50 mL of 25 μM FITC using Lys-peptide-npp resin.
